# Systematic Inspection of the Eye*Abstract of Lecture delivered at New York Polyclinic, July 30th. From notes by Dr. F. L. Oswald, Clinical Assistant.

**Published:** 1896-12

**Authors:** H. E. Stafford

**Affiliations:** New York, Adjunct Professor of Ophthalmology, New York Polyclinic


### SYSTEMATIC INSPECTION OF THE EYE.*

By H. E. STAFFORD, M.D., NEW YORK.,

Adjunct Professor of Ophthalmology, New York Polyclinic.

GENTLEMEN:—As many new faces are in the class to-day, I shall devote part of this lecture to a consideration of the routine inspection of the eye.

It is well to proceed from without inward. With the patient in a good light, before touching the eyes or lids, note the form, color, and mobility of the lids, the width and length of the palpebral fissure, the position and appearance of the eyelashes, any evidences of discharge, and any redness of the exposed globe. Inspect the lachrymal sac at the nasal corner of the eye, pressing it with the fingers, and at the same time note any discharge and the character and consistency of it. Next, test the tension of the ball. Direct the patient to look down and close the lids. Place the tips of the two index-fingers on the upper lid just above the cartilage and press alternately just as in feeling for fluctuation in a swelling. It is necessary to know, of course, the tension of the normal eye. Normal tension is usually designated by T; a slight increase by T + 1; a greater increase by T + 2; and a stony hardness by T + 3. A perceptible softening is represented by T — 1; more decided softening by T — 2; and a complete loss of tension by T — 3.

Now separate the lids gently—use no force. Notice the puncta lachrymalia, upper and lower, their position and appearances. Evert the lids. The lower is everted by simply pulling the lid down and asking the patient to look up. This exposes the lower conjunctiva. To evert the upper lid request the patient to look down, then grasp the eyelashes growing from about the middle of the lid, and with a pen-handle or match-stick or finger of other hand making a little downward pressure about half an inch from the free border of the lid, draw the lid gently downward, forward, and then upward; the torsal cartilage will thus be made to fold over and

*Abstract of Lecture delivered at New York Polyclinic, July 30th. From notes by Dr. F. L. Oswald, Clinical Assistant.

the upper conjunctival surface is exposed. This maneuver is usually necessary in searching for foreign bodies in the eye. Very often a foreign body will be found adhering to the inner surface of the upper lid. Small bodies which have become fixed beneath the lower lid are almost invariably near its tarsal margin. Having everted the lids notice the redness, the degree of swelling and character of the conjunctival and subconjunctival tissues. Note any cicatrices (remains of old trachoma), new growths and any changes in color. Normally these tissues are transparent, and we can readily see through them and the little yellowish striæ that mark the position of the Meibomian glands and ducts. A simple redness or hyperemia of the conjunctiva, which gives rise to the so-called bloodshot appearance, will reveal, on close inspection, an enlargement and prominence of the vessels on the inside of the lid. This condition is usually caused by foreign bodies, smoke or dust, eye-strain, excessive weeping or exposure of the eye to the glare of the sun on snow or white sand, etc. Subjective symptoms are photophobia, lachrymation, and a smarting sensation. The redness due to inflammation of the conjunctiva is generally most prominent on the inner surface of the lids, becoming less as the conjunctiva passes over to the globe and still less near the margin of the cornea, differing thus from the peri-corneal hyperemia which comes from lesions of the cornea, iris, and ciliary body. When the latter become inflamed their enlargement causes the appearance of a pinkish zone, most deeply colored at the corneal margin and shading off gradually into that of the white sclera. It should be remembered that both kinds of hyperemia may exist together.

Swelling of the conjunctiva may be a general thickening, as in acute or chronic conjunctivitis; in the former there is a true exudation into the tissues, the latter is more of a hyperplasia; or there may be only an unevenness of the surface, as in granular or chronic conjunctivitis.

Next examine carefully the cornea, iris, and lens. This is best done in a good light under oblique illumination by means of a convex lens whose focal distance is about two inches. This may be used in ordinary daylight, or still better by gaslight in a dark room. The lens is held about two inches from the eye, condensing the light on one side of it while the observer looks from the other. Notice any hyperemia, and whether it be superficial, as in conjunctivitis, or deep, as in the irregular purple patches of a scleritis. Notice any hemorrhages, likewise any phlyctenular spots consisting of small elevations with an abraded surface surrounded by a zone of hyperemia.

*The pupil* should be tested as to its size, clearness, and mobility, and a comparison made with the opposite pupil. Note the color and contractility of the iris (the latter being tested by placing the hand close to eye for a moment and then suddenly withdrawing it, allowing a good light to fall upon the eye). Notice any irregularity in the shape of the pupil, and whether the iris is adherent to the cornea (anterior synechia), or to the capsule of the lens (posterior synechia). Compare the two eyes and see if both irides are alike. When in doubt about the existence of iris adhesions the instillation of atropine will settle the question.

*The Cornea.*—The healthy cornea is of the most perfect transparency, and its surface smooth and brilliant. These qualities are lost as soon as the part becomes inflamed, a general haziness overspreads the whole surface, and it looks more or less like ground glass. If a healthy eye be examined in front of a window, a perfect image of the window frame should be reflected from the corneal surface. Even a slight inflammation of the cornea, just enough to produce thickening of epithelium, destroys this smooth reflecting surface and causes the window image to appear dull and blurred and the lines of the sash frame wavy and crooked. This appearance is often important in making an early diagnosis of inflammation of the cornea. It may also be seen in the early stages of glaucoma. Examine for opacities and ulcers of the cornea. Note any alteration in its shape and curvature. Ascertain if the cornea or conjunctiva, if touched by a fleck of cotton or hair, exhibit their proper sensibility.

Be sure to notice the depth of the anterior chamber. A shallow anterior chamber is always indicative of glaucoma, unless it be due to a loss of the aqueous humor. In glaucoma we have also a dilated pupil, resembling in this respect a serous iritis, but differing from the latter in having a shallow anterior chamber. Examine carefully, under oblique illumination, for pus in the anterior chamber (hypopyon).

*The crystalline lens* should receive especial care. Ascertain whether the lens be clear, smoky, or positively opaque. As all opacities of the lens are of the nature of a cataract, you will see the importance of being able to diagnose it correctly and of telling your patient the proper time for its extraction. This examination is best made by direct and oblique illumination in a dark room. The pupil should be fully dilated with atropine.

Notice the mobility of the eyes by requesting the patient to watch and follow your finger as this is moved in different directions. Thus paralysis of any muscle may be detected.

It may be impossible at one sitting to accomplish all of what I have proposed above on account of pain, or fear, or photophobia, or refusal of the patient to submit to a complete examination. Pain and photophobia may be obviated by the use of cocaine, four-per-cent. solution. With children an anesthetic may be necessary.

For examination of the posterior parts of the eye the ophthalmoscope will be necessary. Description of its use will be deferred till another lecture.

